# Comparative Effects of Weighted Vest and Whole-Body Vibration Training on Bone and Muscle Health in Osteopenia

**DOI:** 10.3390/life16020229

**Published:** 2026-02-01

**Authors:** Jidapa Tantanasest, Apiwan Manimmanakorn, Orathai Tunkamnerdthai, Chiraphorn Khaengkhan, Peeraporn Nithisup, Suphawijak Youdprang, Nuttaset Manimmanakorn, Michael John Hamlin

**Affiliations:** 1 Exercise and Sport Sciences Program, Multidisciplinary Program, Graduate School, Khon Kaen University, Khon Kaen 40002, Thailand; jidapatan@kkumail.com (J.T.); suphawijakyoudprang@kkumail.com (S.Y.); 2 Department of Physiology, Faculty of Medicine, Khon Kaen University, Khon Kaen 40002, Thailand; torata@kku.ac.th (O.T.); aji_kka@hotmail.com (C.K.); 3 Department of Physical Therapy, Faculty of Allied Health Sciences, Nakhon Ratchasima College, Nakhon Ratchasima 30000, Thailand; peerni@nmc.ac.th; 4 Department of Rehabilitation Medicine, Faculty of Medicine, Khon Kaen University, Khon Kaen 40002, Thailand; natman@kku.ac.th; 5 Department of Tourism, Sport and Society, Faculty of Environment, Society & Design, Lincoln University, Lincoln 7608, New Zealand; michael.hamlin@lincoln.ac.nz

**Keywords:** older adults, bone mineral density, osteoporosis, T-score

## Abstract

Background: The growing elderly population faces health problems like osteoporosis, but novel exercises like weighted vests (WV) and whole-body vibration (WBV) may help prevent bone loss. Methods: Thirty-one women aged 60–79 years with osteopenia or osteoporosis (T-score −2.15 ± 0.9) were randomly assigned to three groups: a control group performed exercise only; a whole-body vibration group performed the exercise on a 40 Hz, 2 mm vibration platform; and a weighted vest group performed the exercise while wearing a weighted vest. T-score, bone mineral density (BMD), muscle mass, and physical performance were assessed before and after 8 weeks. Results: The WV showed a greater T-score increase than the CT and WBV groups (WV: 0.08 ± 0.03; CT: −0.18 ± 0.04; WBV: −0.11 ± 0.16; *p* = 0.01, 95%CI). Leg BMD increased in the WV group (1.75 ± 0.13 to 1.79 ± 0.16 g/cm^2^; *p* = 0.02). Leg lean mass also increased in the WV (1.28 ± 0.91 kg) compared to WBV (0.17 ± 0.14 kg) and CT (0.06 ± 0.79 kg, *p* = 0.01, 95%CI). The WV group showed greater physical performance improvements (5TSTS and 6-MWT). The WBV group showed improved total lean mass compared to the CT group (WBV: 0.32 ± 0.17; CT = −1.20 ± 1.86, *p* = 0.006, 95%CI). Conclusions: WV exercise improved bone density, leg lean mass, and physical performance in older women. WBV exercise increased total lean mass and skeletal muscle index while reducing fat mass. WV exercise provides an additive effect beyond exercise alone or WBV.

## 1. Introduction

The most prevalent health concerns for older adults are conditions characterized by bone mass loss, such as osteopenia and osteoporosis [1,2]. Bone loss progresses with age, estimated at 6.0% annually for those aged 60–69, 1.1% for those aged 70–79, and 1.2% for individuals over 80 [3]. Bone mineral density (BMD) is a crucial indicator of osteopenia and osteoporosis in older adults. Dual-energy X-ray absorptiometry (DXA) is the standard method for measuring BMD, and the World Health Organization (WHO) guidelines use the T-score as a diagnostic criterion. Osteoporosis is diagnosed with a BMD T-score of ≤−2.5, whereas osteopenia is indicated by a T-score between −1.0 and −2.5 [4]. The incidence of osteopenia is higher in women (47.9%) than in men (39.9%), and osteoporosis affects 15.5% of women and 12.8% of men [5]. Another common issue in older adults is a decline in muscle mass, known as sarcopenia. This is caused by a reduction in the number and size of muscle fibers, inactivity, poor nutrition, or nerve damage, leading to muscle atrophy which impairs strength and physical function [6]. Furthermore, older adults tend to experience an increase in fat mass, including fat accumulation within muscle tissue [7,8]. Notably, previous studies have linked reduced muscle strength and mass to a higher prevalence of low BMD and osteoporosis. Loss of bone mineral density increases the risk of disability, mortality, and a decreased quality of life [5].

Regular exercise has been shown to improve bone mass and strength in older adults; however, achieving the necessary long-term training can be challenging in this population. Resistance exercise is recommended as an effective method for musculoskeletal conditions to stimulate bone formation (osteogenesis) in individuals with osteoporosis [3]. This type of exercise can enhance muscle strength, bone mass, balance, walking speed, fall prevention, functional mobility, and ability to perform daily activities in the older adults [9]. However, traditional resistance exercises often involve heavy loads, which may increase the risk of muscle, tendon, and ligament injuries, making them potentially unsuitable for older adults [5,10]. Whole-body vibration training and weighted vest training are emerging techniques that have demonstrated effectiveness in improving balance, muscle strength, bone mass, physical fitness, and reducing the risk of falls in older adults [11,12,13].

Weight-bearing exercise can enhance mobility, bone strength, physical function, prevent fractures, and reduce falls, and weight training can effectively increase bone mineral density (BMD) [14]. Light exercise combined with a weight vest was recommended as a suitable exercise for older adults and reported that 8-week exercise program can improve bone and muscle mass in the elderly [15]. This type of exercise generates greater joint reaction forces than those experienced during typical daily activity. Weight-bearing training is often well received by older individuals because of its harmless nature, self-management potential, and ease of practice [16]. Using a weighted vest worn on the upper body increased the weight-bearing load and intensity of training. Combining a weighted vest with exercise may enhance muscle contraction, metabolic cost, and the load on joints, bones, and muscles [17]. Weighted vest training has been employed as an intervention to improve muscle strength and physical performance in the elderly population [18,19,20,21]. The effectiveness of weighted vest training may be related to the added load intensity. Previous research has suggested that a 20% body weight load might be excessive for older adults, while a 3–5% load might be insufficient to improve performance [22]. However, another study found that a 10% body weight load resulted in no injuries and led to improvements in strength, sit-to-stand performance, and aerobic capacity compared with exercise alone [16]. Furthermore, weighted vest training can improve the bone density in individuals with osteoporosis [23,24]. A study by Tantiwiboonchai et al. (2011) demonstrated that a 30 min walking exercise on a treadmill with an 8% bodyweight weighted vest, three times per week for 12 weeks, could reduce bone resorption and improve physical fitness [25].

Whole-body vibration training is a novel, non-invasive, and non-pharmacological neuromuscular intervention that can promote bone density and muscle mass, thereby improving balance, mobility, gait performance, muscle strength, reducing the risk of falls, and preventing osteoporosis [26,27,28,29]. This training method generates mechanical forces through oscillating platforms that are then transferred to the weight-bearing bones of the skeleton. WBV stimulates muscle contractions via the tonic vibration reflex, in which vibration waves stimulate muscle spindles, leading to increased muscle activity and power [30]. Previous studies have indicated that whole-body vibration training (with frequencies ranging from 2 Hz to 90 Hz and amplitudes from 0.7 mm to 12 mm) appears more effective in improving bone and muscle strength in postmenopausal women than walking alone [14]. Consequently, numerous studies have explored the use of whole-body vibration training to manage osteopenia and sarcopenia in the elderly, with results showing improvements in muscle strength, bone mass, fall reduction, balance, gait, and overall quality of life [13,26].

Despite their potential, there is currently a lack of research and clinical data regarding the use of whole-body vibration and weighted vest training as therapeutic stimuli for elderly patients with osteopenia. Specifically, the effectiveness of short-duration alternative exercises for these two methods remains under-explored in this demographic. Investigating these combined approaches could establish a practical and efficient strategy for clinical interventions in older adults. This study investigated whether adding weighted vest training or whole-body vibration to a light exercise regimen enhances bone density, lean mass, and physical performance in elderly individuals with osteopenia. It was hypothesized that both interventions would yield superior results compared to light exercise alone, with weighted vest training expected to demonstrate a greater impact on musculoskeletal health than whole-body vibration.

## 2. Materials and Methods

### 2.1. Study Design

**Figure 1:**
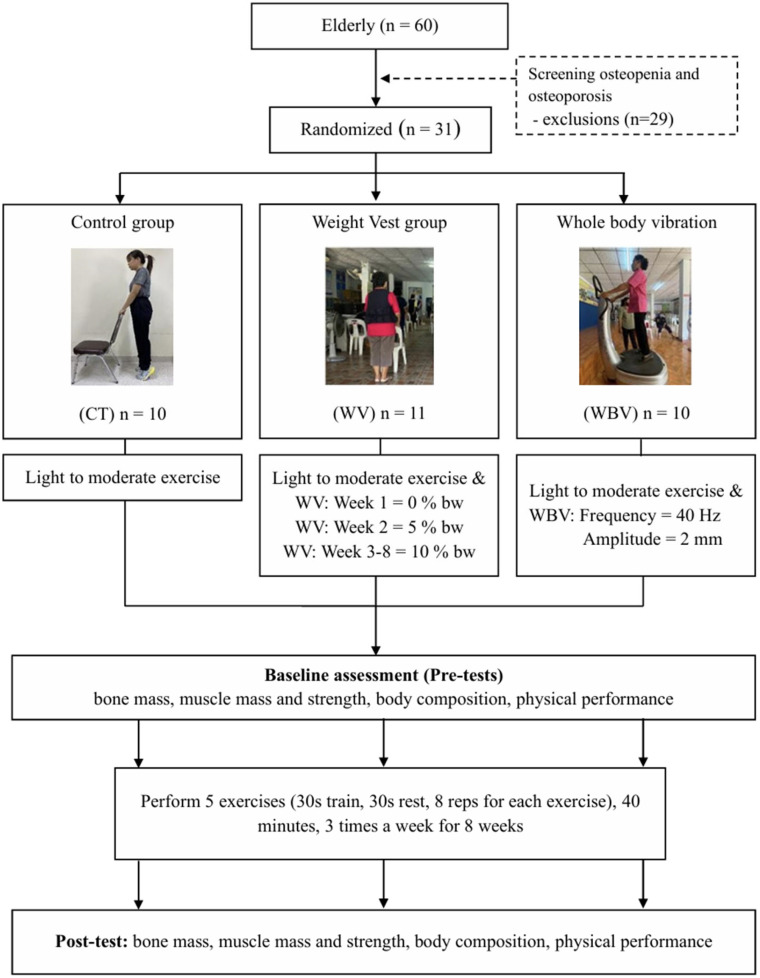
Diagram of the study.

Participants were assigned to one of three groups using computer-generated random sequences, with allocation concealment ensured through sealed envelopes managed by an independent statistician. To ensure baseline comparability, the 31 participants were first ranked by bone mineral density scores before being randomly distributed into their respective groups. Following the 8-week intervention, the final cohort consisted of the control group (CON: *n* = 10), the weighted vest group (WV: *n* = 11), and the whole-body vibration group (WBV: *n* = 10). Throughout the study, subjects were instructed to adhere to their baseline dietary habits and daily activities while refraining from additional exercise (Figure 1).

Sample size estimation for the overall project was conducted using G*Power 3.1, which indicated that 30 participants would provide sufficient power (effect size = 0.93, α = 0.05, power = 0.80) [15]. This manuscript presents an analysis of participants with osteopenia (*n* = 31), derived from the randomized controlled trial.

The Intention-to-Treat (ITT) approach was used as the primary analytical strategy. Since there were no dropouts or protocol deviations during the study period, the ITT population is identical to the per-protocol population. All randomized participants (*n* = 31) completed the intervention and were included in the final analysis.

### 2.2. Participants

Participants were elderly individuals aged 60–79 years. They were required to have been on stable optimal medication for at least six months, with no crises or medication changes for at least three months prior to enrollment. For participants with high blood pressure, their condition had to be stable, with resting systolic blood pressure ≤140 mmHg and diastolic blood pressure ≤90 mmHg, as defined by the 2020 International Society of Hypertension Global Hypertension Practice Guidelines, and diagnosed by a physician. All participants underwent a structured interview and medical history review to screen for the use of bone-affecting medications. Individuals receiving anti-resorptive or anabolic treatments (e.g., bisphosphonates, denosumab, or hormone replacement therapy) within the past six months were excluded to isolate the effects of the exercise intervention.

Inclusion criteria for bone density, based on WHO guidelines, were a T-score between −1.0 and −2.4 for osteopenia and a T-score ≤ −2.5 for osteoporosis [5]. Elderly individuals were excluded from participation if they met any of the following conditions:

- A serious respiratory disorder (e.g., asthma, COPD)
- A history of cardiovascular diseases (e.g., acute myocardial infarction, unstable angina, heart failure, stroke)
- A history of neuromuscular or musculoskeletal diseases lasting more than one month
- Chronic kidney disease (stage I or higher)
- Endocrine disorders (e.g., hyperthyroidism, hypothyroidism)
- Psychological symptoms (e.g., anxiety, depression)

All participants were community-dwelling elderly individuals residing in Khon Kaen province who had been diagnosed with osteopenia or osteoporosis based on T-scores obtained using dual X-ray absorptiometry (DXA), the standard method for measuring bone mineral density (BMD). All participants received complete information about the study’s purpose and provided written informed consent. Thirty-one participants met inclusion criterion, completed exercise program, and then were brought to be analysed.

### 2.3. Ethical Approval

Prior to participation, all individuals were provided with an informed consent document outlining the objectives, procedures, potential benefits, and associated risks of the study. The researcher thoroughly explained the standard informed consent to each participant. Written informed consent was obtained from all participants following a complete explanation of the study procedure and potential risks. Ethical approval for this study was obtained from the Khon Kaen University Research Ethics Committee (HE651481). Clinical trial registration was obtained through the Thai Clinical Trials Registry (TCTR) with the identification number TCTR20240529009.

### 2.4. Intervention

Elderly female participants diagnosed with osteopenia (*n* = 31) were randomly allocated to one of three experimental groups using a drawing method: control group (CT), weighted vest group (WV), and whole-body vibration group (WBV). The CT group engaged in a home-based exercise program, the WBV group performed exercises on a whole-body vibration machine, and the WV group performed exercises while wearing a weighted vest. All groups adhered to an identical exercise regimen consisting of a 10 min warm-up (walking with arm lifts) followed by five distinct exercises (knee bends, tiptoe standing, wide-stance knee bends, right leg forward step with knee flexion, and right leg forward step with bilateral knee flexion). Each exercise was performed for 30 s and repeated for a total of eight sets with 30 s rest periods between sets, resulting in a 40 min exercise session. The program was implemented three times per week over an eight-week period.

Participants in the WBV group were exposed to vibration using a Power Plate Pro 5 Silver (Performance Health Systems LLC, Northbrook, IL, USA) Vibration using a Power Plate Pro 5 Silver (Performance Health Systems LLC, Northbrook, IL, USA). set at a frequency of 40 Hz and an amplitude of 2 mm. The participants stood on the platform without footwear and maintained 20° knee flexion while holding onto a handle.

This study involved assessments of the primary outcomes are bone mass (T-score and BMD), the secondary outcomes are lean mass, fat mass and physical performance. These evaluations were conducted at baseline, one day prior to the commencement of the experimental period, and at post-test, one day after the completion of the eight-week intervention.

### 2.5. Measurements of Parameters Study

#### 2.5.1. Bone Mass and Muscle Mass

Dual X-ray Absorptiometry (DXA), a validated instrument for evaluating bone remodeling, was utilized to measure bone and muscle mass. This technique quantifies the total and regional fat, bone mineral content, and bone mineral-free lean mass based on the differential attenuation of X-rays at two distinct peak energies. For the DXA scan, the participants were positioned supine with their legs supported in a standardized manner to ensure neutral alignment. Consistent with the preparation for body composition analysis, participants were instructed to have a bowel movement within 30 min and to abstain from alcohol for a minimum of 48 h and food for a minimum of 4 h prior to the examination.

#### 2.5.2. Body Composition

Bioelectrical impedance analysis (BIA) was employed to determine body composition, specifically by measuring body weight, body mass index (BMI), lean body mass, body fat mass, and percentage of body fat. The participants were required to remove their socks and stand barefoot on the metallic electrodes of the BIA device. Furthermore, they were instructed to have a bowel movement within 30 min and to avoid alcohol consumption for a minimum of 48 h and food intake for a minimum of 4 h before the assessment.

#### 2.5.3. Single Leg Stance Test (STS)

Static balance was evaluated using the Single Leg Stand (SLS) test, a widely applied measure in geriatric research [31]. Participants were instructed to stand on one leg, with eyes open and arms crossed over the chest, maintaining the position for as long as possible. Test duration was recorded from the moment the foot was lifted until the raised leg shifted, touched the ground, or arm support was used. Three trials were performed with 5 min rest intervals, and the longest time achieved was taken as the participant’s score.

#### 2.5.4. Five Time Sit to Stand (5TSTS) Test

The Five-Time Sit-to-Stand (5TSTS) test was used to assess lower extremity functional strength, transitional movement ability, balance, and fall risk. Participants were instructed to rise from a seated position and return to sitting five consecutive times, with the total completion time recorded in seconds to one decimal place [32].

#### 2.5.5. Balance Test (Timed up and Go: TUG)

Balance was assessed using the Timed Up and Go (TUG) test, a reliable and valid measure in older adults [33]. Participants were instructed to rise from a chair with armrests, walk 3 m at a comfortable pace, turn, return to the chair, and sit. The time taken to complete the task was recorded from the moment of standing until reseating [34].

#### 2.5.6. Endurance Test (Six-Minute Walk Test: 6-MWT)

The Six-Minute Walk Test (6-MWT) was administered to evaluate exercise capacity in older adults by measuring the total distance walked within six minutes. A 30 m linear course was marked with cones, with a chair placed at the midpoint for optional rest. Participants began walking at a self-selected pace and continued for six minutes. At test completion, the total distance and elapsed time were recorded. The test was terminated if participants reported a rating of perceived exertion (RPE) greater than 8 or requested to stop [35].

### 2.6. Statistical Analyses

Descriptive statistics were expressed as mean ± standard deviation (mean ± SD) for baseline, pre-test, and post-test values. The normality of the data was evaluated using the Shapiro–Wilk test. Baseline comparisons among the three groups were conducted using one-way analysis of variance (ANOVA). Within-group differences between pre- and post-intervention values were assessed using paired *t*-tests. Between-group differences in change scores were analyzed using post hoc comparisons when significant main effects were identified. For the change scores (post–pre differences), data were presented as mean ± 95% confidence intervals. Statistical significance was established at *p* < 0.05.

## 3. Results

### 3.1. Clinical Characteristics and Physiological Variables at Baseline

At baseline, no statistically significant differences in clinical characteristics or physiological variables were observed among the three groups (Table 1). All participants demonstrated high adherence to the exercise program, completing at least 90% of sessions without reporting adverse effects.

### 3.2. Effects of Weighted Vest and Whole-Body Vibration Training on Bone Mass Parameters

Table 2 presents key changes in bone mineral density (BMD). In the WV group, significant increases were observed in both leg and total BMD compared with baseline values. Specifically, leg BMD improved by 0.02 ± 0.01 g/cm^2^ (*p* = 0.01), while total BMD increased by 0.01 ± 0.00 g/cm^2^ (*p* = 0.05), expressed as mean ± 95%CI. In contrast, the CT group showed a significant decline in total BMD relative to baseline, with a mean change of –0.01 ± 0.02 g/cm^2^ (*p* = 0.001; mean ± 95%CI).

Regarding T-scores, the WV group exhibited a significant increase compared with both the CT and WBV groups. The mean change in T-score was 0.08 ± 0.03 for the WV group, whereas the CT and WBV groups showed reductions of –0.18 ± 0.21 and –0.11 ± 0.24, respectively (*p* = 0.01; mean ± 95%CI) (Table 2).

### 3.3. Effects of Weighted Vest and Whole-Body Vibration Training on Muscle Mass Parameters

Following the training period, the WV group demonstrated a significant increase in Leg lean mass compared with both the CT and WBV groups. The mean change was 1.28 ± 0.91 kg in the WV group, whereas the CT and WBV groups showed minimal changes of 0.06 ± 0.79 kg and 0.17 ± 0.14 kg, respectively (*p* = 0.001; mean ± 95%CI) (Table 3). With respect to total lean mass, both the WV and WBV groups demonstrated statistically significant changes compared with the CT group. The CT group showed a reduction of –1.20 ± 0.98 kg, whereas the WV group exhibited a smaller decrease (–0.23 ± 0.68 kg) and the WBV group showed a modest increase (0.32 ± 0.73 kg) (*p* = 0.006; mean ± 95%CI). Regarding the Skeletal Muscle Index (SMI), the WBV group demonstrated a significant improvement compared with the CT group. The WBV group showed a mean change of 0.23 ± 0.05, whereas the CT group exhibited a reduction of –0.21 ± 0.39 (*p* = 0.02; mean ± 95%CI) (Table 3).

### 3.4. Effects of Whole-Body Vibration Training and Weighted Vest Training on Fat Mass Parameters

Surprisingly, the WBV group demonstrated a substantial reduction in fat mass parameters following the training intervention, including trunk fat mass, total fat mass, and % body fat (*p* < 0.05) (Table 3). Notably, the WBV group showed a significant decrease in % body fat compared with both the CT and WV groups. The mean change in % body fat was –0.74 ± 1.88% for the WBV group, whereas increases were observed in the CT (1.13 ± 0.62%) and WV (1.01 ± 0.34%) groups (*p* = 0.001; mean ± 95%CI). Unexpectedly, the WV group exhibited a significant reduction in leg fat mass compared with baseline values (*p* = 0.001). Moreover, this decrease was significantly greater than that observed in both the CT and WBV groups. The mean change in leg fat mass was –1.85 ± 0.61 kg in the WV group, compared with 0.17 ± 0.01 kg in the CT group and –0.11 ± 1.04 kg in the WBV group (*p* = 0.001; mean ± 95%CI) (Table 3).

### 3.5. Effects of Whole-Body Vibration Training and Weighted Vest Training on Physical Performance Parameters

Both the WV and WBV groups demonstrated significant improvements in Timed Up and Go (TUG), Single Leg Stand (SLS), Five-Time Sit-to-Stand (5TSTS), and Six-Minute Walk Test (6-MWT) compared with their respective baselines. When comparing mean changes between groups, the WV group showed a significantly greater improvement in 5TSTS than the other groups (WV = −6.32 ± 2.55 s; WBV = −3.60 ± 5.31 s; CT = −3.02 ± 4.48 s; *p* = 0.001, 95%CI). In addition, the WV group exhibited a substantial increase in 6-MWT performance compared with the WBV and CT groups (WV = 100.75 ± 41.61 m; WBV = 50.27 ± 27.10 m; CT = 20.38 ± 4.08 m; *p* = 0.010, 95%CI) (Table 4).

## 4. Discussion

This study examined the effects of eight-week program involving either weighted vest (WV) training or whole-body vibration (WBV), both paired with light exercise, on bone mineral density, muscle mass, and physical performance in elderly women with osteopenia. The key findings revealed that WV training combined with light exercise led to greater gains in leg bone density, T-score, leg lean mass, and physical performance (measured by the 5TSTS and 6-MWT). While WBV group demonstrated improvements in total lean muscle mass and reduction in fat mass.

Bone mineral density, T-score, and Z-score: The findings revealed that participants in the weighted vest (WV) group showed a marked improvement in both leg BMD and total BMD relative to baseline values. Notably, their T-scores increased more than those observed in the other groups. This indicates that adding external weight during exercise is more effective for enhancing leg bone mass compared to exercise alone. The results further highlight that even a modest load (10% of body weight) can provide a protective benefit against bone loss, particularly in the leg region. In contrast, light exercise by itself did not demonstrate a protective effect, with bone mass gradually declining over the eight-week period (Table 2). The notable gains in leg bone mineral density (BMD) and T-score observed in the weighted vest (WV) group align with earlier studies that employed weight-bearing exercise or resistance training. Kelley et al. (2012) demonstrated that exercises involving ground and joint reaction forces can enhance bone density at the lumbar spine and femoral neck after a minimum of 24 weeks of training [36]. Similarly, Zehnacker and Bemis-Dougherty (2007) reported that weighted exercise contributes to maintaining bone mineral density (BMD) in the spine and hip among older adult with osteoporosis [37], while also improving strength, balance, and reducing fall risk [38]. Hakestad et al. (2015) reported increased leg BMD in elderly women with osteopenia after six months of WV training (4–6% body weight) combined with a rehabilitation program [39]. Interestingly, the current research employed a shorter training duration, specifically an eight-week program. Previous studies reported that although the complete human bone remodeling cycle typically spans approximately 200 days [40], our findings suggest that the heightened mechanical strain provided by weighted vests may trigger accelerated bone modeling and rapid mineral apposition [41]. By suppressing sclerostin and enhancing osteoblast activity, this intervention may increase the mineral density of existing trabeculae before a full turnover cycle is completed [42]. Longer-term studies are necessary to confirm sustained effects.

Lean mass and Skeletal Muscle Index (SMI): Whole-body vibration (WBV) training combined with light exercise produced significant improvements in total lean mass and skeletal muscle index (SMI) (Table 3). Machado et al. (2009) reported that 10 weeks of WBV training with squats increased knee and hip extensor muscle mass and strength [43]. Similarly, Lau et al. (2011) found that WBV enhanced muscle mass and strength, though it did not consistently improve bone mass in elderly women [44]. In contrast, Perchthaler et al. (2015) observed no significant gains in leg muscle strength among older adults after six weeks of WBV training with dynamic squats [45]. The underlying mechanism for WBV’s effect on muscle strength is thought to involve the vibratory tonic reflex, which activates muscle spindles and enhances muscle recruitment [46]. WBV has been shown to stimulate EMG activity in both trunk and limb muscles [45,47], and repeated exposure can induce neuromuscular adaptations such as hypertrophy and hormonal changes [43], ultimately improving strength and balance. Moreover, WBV may increase blood flow and oxygen uptake, support recovery [48], while elevating heart rate during exercise, making it a useful warm-up modality. The observed rise in SMI in the WBV group reinforces the additive benefits of combining WBV with exercise to enhance muscle mass in elderly individuals with osteopenia.

Otherwise, whole body vibration training in older adults, Weber-Rajek et al. highlighted improvements in muscle strength and balance [49]. Similarly, randomized trials have shown weighted vest exercise to reduce bone resorption and enhance physical fitness. More recently, vibration-based interventions were reported to improve bone quality and muscle function in postmenopausal women [50]. These studies support the potential of both modalities as practical, non-pharmacological strategies for osteopenia management.

Fat mass: Whole-body vibration (WBV) training led to reductions in trunk fat, total fat mass, and body fat percentage following the exercise program, this is some unexpected outcome. In contrast, weighted vest (WV) exercise was most effective in decreasing fat mass specifically in the legs. These findings suggest that WBV may enhance muscle activation in both trunk and limb regions, thereby increasing oxygen consumption and promoting the utilization of fat tissue in leg muscles as an energy source. Similarly, the reduction in leg fat mass observed with weighted vest (WV) training may be attributed to the mechanical load applied to the leg muscles and increased the mechanical demand on the knee and hip extensors and ankle plantar flexors [20], which is accompanied by increases in leg muscle mass. This combined effect likely provides a stronger stimulus in the leg region compared to other areas, resulting in localized fat reduction.

Physical Performance: The exercise program produced positive outcomes across all groups, but the weight vest (WV) group showed distinctly greater benefits, particularly in the 5TSTS and 6-MWT assessments (Table 4). These improvements are linked to the observed increases in bone mass and lean mass following WV exercise. Supporting this, Mierzwicki et al. (2019) reported that 12 weeks of WV training at 10% of body weight combined with low-intensity exercise enhanced muscle strength as well as 5TSTS and 6-MWT performance in older adults [16]. The likely mechanism behind WV training involves heightened muscle activation and elevated energy expenditure [47,51].

### 4.1. Limitations

This study has several limitations. First, participants were not separated by diagnosis (osteopenia vs. osteoporosis), preventing condition-specific evaluation of intervention effects. Second, dietary intake was uncontrolled, as participants maintained their usual eating habits, which may have introduced variability. Future studies should include distinct diagnostic groups and larger sample sizes to improve statistical power. Finally, the relatively short training duration may have provided insufficient stimulus to fully optimize exercise benefits.

### 4.2. Futures Research

To broaden understanding, future studies should include participants with vitamin D deficiency and those in early menopause. Additionally, research should explore the combined effects of weighted vest training and whole-body vibration on bone health to assess their potential synergy in managing these common conditions.

## 5. Conclusions

Weight vest training produced notable gains in bone density, leg lean mass, and physical performance (5TSTS and 6MWT). WBV demonstrated efficacy in enhancing total lean mass and skeletal muscle index, accompanied by a reduction in fat mass. The findings suggest that weighted vest exercise is a viable intervention that provides an additive effect, demonstrating superiority over both exercises alone and the WBV approach.

## Figures and Tables

**Table 1:** Clinical characteristics and physiological variables.

	CT (*n* = 10)	WV (*n* = 11)	WBV (*n* = 10)	*p*-Value
Age (y)	66.00 ± 5.22	69.56 ± 5.61	67.11 ± 5.37	0.485
Heart rate (bpm)	77.56 ± 9.58	75.22 ± 7.66	71.78 ± 9.90	0.413
Systolic blood pressure (mmHg)	129.89 ± 11.04	137.56 ± 9.86	126.67 ± 8.17	0.071
Diastolic blood pressure (mmHg)	73.44 ± 8.99	78.11 ± 7.57	71.22 ± 7.51	0.201
Height (cm)	151.67 ± 4.87	151.44 ± 3.00	155.78 ± 6.30	0.131
Weight (kg)	56.63 ± 9.41	58.12 ± 7.57	58.78 ± 10.68	0.883
BMI (kg/m^2^)	24.27 ± 3.71	24.93 ± 3.29	23.83 ± 3.83	0.811
Waist circumference (cm)	81.98 ± 14.51	91.27 ± 10.51	89.89 ± 12.09	0.254
Hip circumference (cm)	95.19 ± 7.12	98.10 ± 7.19	96.33 ± 7.83	0.705
Waist to Hip ratio (cm)	0.86 ± 0.12	0.93 ± 0.07	0.94 ± 0.14	0.311
Total BMD (g/cm^2^)	0.96 ± 0.06	0.94 ± 0.09	0.96 ± 0.09	0.726
T-score	−2.11 ± 0.83	−2.51 ± 1.24	−1.72 ± 0.79	0.154
Z-score	−0.69 ± 0.42	−0.91 ± 0.93	−7.89 ± 0.82	0.855
Lean mass (kg)	37.07 ± 5.43	35.39 ± 3.86	36.04 ± 4.16	0.819
Fat mass (kg)	20.31 ± 5.54	21.31 ± 4.44	23.26 ± 6.77	0.593
Osteoporosis	2	2	2	
Osteopenia	8	9	8	

Data are presented as mean ± SD, Control (CT, *n* = 10), weighted vest (WV, *n* = 11), Whole body vibration WBV (*n* = 10), BMI = Body mass index.

**Table 2:** Changes bone mineral density (BMD), T-score and Z-score in the three groups before and after 8 weeks of training.

BMD (g/cm^2^)		CT (*n* = 10)	Mean Difference ±95%CI	WV (*n* = 11)	Mean Difference ±95%CI	WBV (*n* = 10)	Mean Difference ±95%CI	*p*-Value
L-spine	Pre Post	0.78 ± 0.08 0.78 ± 0.08	0.00 ± 0.02	0.79 ± 0.17 0.76 ± 0.14	−0.03 ± 0.07	0.81 ± 0.10 0.80 ± 0.07	−0.01 ± 0.05	0.333
Legs	Pre Post	0.90 ± 0.06 0.91 ± 0.07	0.01 ± 0.01	0.89 ± 0.07 0.91 ± 0.08	0.02 ± 0.01 *	0.91 ± 0.07 0.92 ± 0.08	0.01 ± 0.02	0.086
Total	Pre Post	0.96 ± 0.06 0.95 ± 0.06	−0.01 ± 0.02 *	0.94 ± 0.09 0.95 ± 0.09	0.01 ± 0.00 *	0.96 ± 0.09 0.95 ± 0.07	−0.01 ± 0.03	0.655
T-score	Pre Post	−2.11 ± 0.83 −2.29 ± 0.84	−0.18 ± 0.21 *	−2.51 ± 1.24 −2.43 ± 1.25	0.08 ± 0.03 ^a,b^	−1.72 ± 0.79 −1.83 ± 0.79	−0.11 ± 0.4	0.010

Data are mean ± SD for pre-test and post-test and mean ± 95% confidence interval for post-pretest changes. WV = weighted vest, WBV = whole body vibration, CT = control group. * significant difference within group, ^a^ significant difference between CT and WV, ^b^ significant difference between WV and WBV.

**Table 3:** Changes in lean mass and fat mass of participants in the three groups before and after eight weeks of training.

Parameters		CT (*n* = 10)	Mean Difference ±95%CI	WV (*n* = 11)	Mean Difference ±95%CI	WBV (*n* = 10)	Mean Difference ±95%CI	*p*-Value Between 3 Groups
Lean mass (kg)								
Trunk	Pre Post	19.38 ± 3.61 18.35 ± 3.03	−1.02 ± 1.70 *	17.53 ± 1.76 17.03 ± 1.54	−0.31 ± 0.71	17.80 ± 1.95 17.97 ± 2.08	0.16 ± 0.21	0.144
Legs	Pre Post	10.92 ± 1.88 10.98 ± 1.53	0.06 ± 0.79	11.67 ± 2.84 12.94 ± 1.14	1.28 ± 0.91 *^,a,b^	11.74 ± 1.88 11.91 ± 1.92	0.17 ± 0.14	0.001
Total	Pre Post	37.07 ± 5.43 35.87 ± 5.07	−1.20 ± 1.86 *	35.39 ± 3.86 34.50 ± 3.39	−0.23 ± 0.71 ^a^	36.04 ± 4.16 36.37 ± 4.01	0.32 ± 0.17 ^c^	0.006
SMI (kg/cm^2^)	Pre Post	6.43 ± 0.59 6.22 ± 0.64	−0.21 ± 0.39	6.39 ± 0.77 6.31 ± 0.73	−0.08 ± 0.25	6.16 ± 0.73 6.39 ± 0.69	0.23 ± 0.05 ^c^	0.020
Fat mass (kg)								
Trunk	Pre Post	9.80 ± 3.30 10.02 ± 3.15	0.22 ± 0.18	9.45 ± 2.75 9.62 ± 2.91	0.17 ± 0.05	10.59 ± 3.51 9.94 ± 3.75	−0.66 ± 1.27 *^,b,c^	0.005
Legs	Pre Post	6.67 ± 1.37 6.84 ± 1.38	0.17 ± 0.01	9.90 ± 1.58 8.05 ± 2.10	−1.85 ± 0.61 *^,a,b^	8.49 ± 2.53 8.39 ± 2.48	−0.11 ± 1.04	0.001
Total	Pre Post	20.31 ± 5.54 20.71 ± 5.24	0.40 ± 0.20	21.31 ± 4.44 21.45 ± 5.16	0.15 ± 0.72	23.26 ± 6.77 22.48 ± 6.00	−0.78 ± 2.03 ^b,c^	0.035
% Fat	Pre Post	33.98 ± 5.01 35.11 ± 4.52	1.13 ± 0.62 *	35.01 ± 3.37 36.02 ± 4.06	1.01 ± 0.34 *	37.58 ± 5.90 36.83 ± 5.07	−0.74 ± 1.88 ^b,c^	0.001

Data are mean ± SD for pre-test and post-test and mean ± 95% confidence interval for post-pretest changes. WV = weighted vest, WBV = whole body vibration, CT = control group. SMI = skeletal muscle index, * significant difference within group, ^a^ significant difference between CT and WV, ^b^ significant difference between WV and WBV, ^c^ significant difference between CT and WBV.

**Table 4:** Changes in physical performance in three groups before and after 8 weeks training.

Parameters		CT (*n* = 10)	Mean Difference ±95%CI	WV (*n* = 11)	Mean Difference ±95%CI	WBV (*n* = 10)	Mean Difference ±95%CI	*p*-Value Between 3 Groups
TUG (s)	Pre Post	13.13 ± 1.44 9.22 ± 1.10	−3.92 ± 4.73 *	11.79 ± 2.03 6.66 ± 9.85	−5.13 ± 7.16 *^,b^	9.19 ± 1.62 7.07 ± 0.92	−2.11 ± 3.10 *	0.006
SLS (s)	Pre Post	14.95 ± 9.53 33.32 ± 16.91	18.38 ± 5.61 *	13.95 ± 8.51 42.09 ± 31.05	28.14 ± 6.33 *	19.72 ± 12.84 39.75 ± 22.43	20.04 ± 5.62 *	0.605
5TSTS (s)	Pre Post	16.25 ± 3.36 13.22 ± 2.84	−3.02 ± 4.48 *	15.29 ± 2.85 8.97 ± 1.55	−6.32 ± 8.28 *^,a,b^	12.56 ± 1.78 8.96 ± 1.18	−3.60 ± 5.31 *	0.001
6-MWT (m)	Pre Post	327.27 ± 48.51 347.65 ± 35.31	20.38 ± 4.08	315.83 ± 63.62 416.59 ± 53.37	100.75 ± 68.76 *^,a,b^	386.01 ± 42.24 436.28 ± 78.49	50.27 ± 14.07 *	0.010

Data are mean ± SD for pre-test and post-test and mean ± 95% confidence interval for post-pretest changes. WV = weighted vest, WBV = whole body vibration, CT = control group. * significant difference within group, ^a^ significant difference between CT and WV, ^b^ significant difference between WV and WBV.

## Data Availability

The data presented in this study are available on request from the corresponding author.

## Abbreviations

The following abbreviations are used in this manuscript:

BIA	Bioelectrical impedance analysis
BMD	Bone mineral density
BMI	Body mass index
COPD	Chronic Obstructive Pulmonary Disease
DXA	Dual X-ray absorptiometry
RPE	Rating of perceived exertion
SMI	Skeletal Muscle Index
WBV	Whole-body vibration
WV	Weighted vest
5TSTS	Five Time Sit to Stand
6-MWT	Six-Minute Walk Test
SLS	Single Leg Stand
TUG	Timed Up and Go
